# Phospho-kinase profile of colorectal tumors guides in the selection of multi-kinase inhibitors

**DOI:** 10.18632/oncotarget.5211

**Published:** 2015-09-22

**Authors:** Gemma Serrano-Heras, María Dolores Cuenca-López, Juan Carlos Montero, Verónica Corrales-Sanchez, Jorge Carlos Morales, Luz-Elena Núñez, Francisco Morís, Atanasio Pandiella, Alberto Ocaña

**Affiliations:** ^1^ Translational Research Unit, Albacete University Hospital, Albacete, Spain; ^2^ Cancer Research Center, CSIC-University of Salamanca, Salamanca, Spain; ^3^ EntreChem S.L., Oviedo, Spain

**Keywords:** colon cancer, EC-70124, kinase inhibitors

## Abstract

Protein kinases play a central role in the oncogenesis of colorectal tumors and are attractive druggable targets. Detection of activated kinases within a tumor could open avenues for drug selection and optimization of new kinase inhibitors. By using a phosphokinase arrays with human colorectal tumors we identified activated kinases, including the Epidermal Growth Factor Receptor (EGFR), components of the PI3K/mTOR pathway (AKT and S6), and STAT, among others. A pharmacological screening with kinase inhibitors against these proteins helped us to identify a new kinase inhibitor, termed EC-70124 that showed the highest anti-proliferative activity in cell lines. EC-70124 also inhibited cell migration and biochemical experiments demonstrated its effect targeting the PI3K/mTOR pathway. This drug also arrested cells at G2/M and induced apoptosis. Experiments in combination with standard chemotherapy used in the clinical setting indicated a synergistic effect. EC-70124 also reduced tumor growth *in vivo* and inhibited pS6 in the implanted tumors. In conclusion, by studying the kinase profile of colorectal tumors, we identified relevant activated pathways, and a new multi-kinase compound with significant antitumor properties.

## INTRODUCTION

Different molecular alterations have been described in colorectal cancer. Among them, the unbalanced activation of protein kinases plays a central role [1]. Several of these proteins, including receptor tyrosine kinases (RTK) or signaling downstream mediators, have been associated with the initiation, maintenance and progression of this tumor type [1]. An example is the expression of the Epidermal Growth Factor Receptor (EGFR), and the Vascular Endothelial Growth Factor Receptor (VEGFR) in colorectal cancer, that led to the clinical development of drugs against them, such as panitumumab or cetuximab against EGFR, and bevacizumab against VEGFR [1, 2]. This activation is also associated with an oncogenic advantage, as pharmacological inhibition with the mentioned compounds is linked with clinical benefit [3, 4].

Taken into account that solid tumors, and particularly colorectal cancer, is a heterogeneous disease [2], the understanding of the kinase profile of this tumor could help in the selection of relevant therapeutic strategies. This approach has been used previously to identify the PI3K/mTOR route as a relevant target in a subtype of breast tumors [5]. In addition, the increase therapeutic efficacy observed when acting concomitantly against several kinases compared with single kinase inhibition, suggests that the identification, selection, and therapeutic optimization of inhibitors with a broader effect on relevant proteins kinases can represent a better therapeutic strategy, if there is no increase in toxicity [6].

In this regard, several proteins and signaling routes are clearly activated in colon cancer and linked with tumorigenesis. Some of them include the PI3K/mTOR pathway, the Mitogen Activated Protein Kinase (MAPK) route, angiogenesis pathways or routes associated with migration such as the FAK family of kinases [7, 8]. In parallel with this, some of these routes have been linked with resistance to targeted therapies against known oncogenes reinforcing the concept that a global kinase picture could undoubtedly provide useful information [9]. Therefore, a desirable approach would be the development of polypharmacology inhibitors targeting simultaneously several of these relevant pathways and proteins.

In the present work, we planned to explore the kinase profile of primary colorectal tumors; and based on these findings, to perform a pharmacologic screening to recognize kinase inhibitors with anti-proliferative effect. We identified a novel compound termed EC-70124 with an ample inhibitory spectrum including the PI3K/mTOR pathway and SRC. EC-70124 showed inhibition of proliferation and migration in preclinical models; and tumor growth inhibitory properties in animals. Furthermore, this compound induced DNA damage and synergized with chemotherapy used in the clinical setting. Taken together this data support the future clinical evaluation of this compound in colorectal tumors.

## RESULTS

### Phospho-kinase profile of human colorectal tumors

We analyzed the activation status of several RTKs and relevant signaling mediators in samples from eighteen patients diagnosed with colorectal cancer. To do so, we used two antibody-based array kits that evaluate the phosphorylation status of these proteins, as shown in Supplementary Figure S1. Patient characteristics are described in Table 1.

**Table 1:** Patient and tumor characteristics

Patient age (*n* = 18)		Median 73,5 (range 54–84)
Tumor type (*n* = 18)	Adenocarcinoma	14 (78%)
Patients *n* = 16	Positive	7 (43,75%)
	Negative	9 (56,25%)
pT (*n* = 18)	1	1 (5,6%)
	2	4 (22,2%)
	3	10 (55,5)
	4	3 (16,6)
pN (*n* = 18)	0	9 (50%)
	1	4 (22,2%)
	1a	1 (5,6%)
	2	3 (16,6%)
	2b	1 (5,6%)
M (*n* = 18)	0	13 (72%)
	1	5 (28%)
Kras Mutated (*n* = 18)	Positive	8 (44,4%)
	Negative	10 (55,6%)

**Figure 1:**
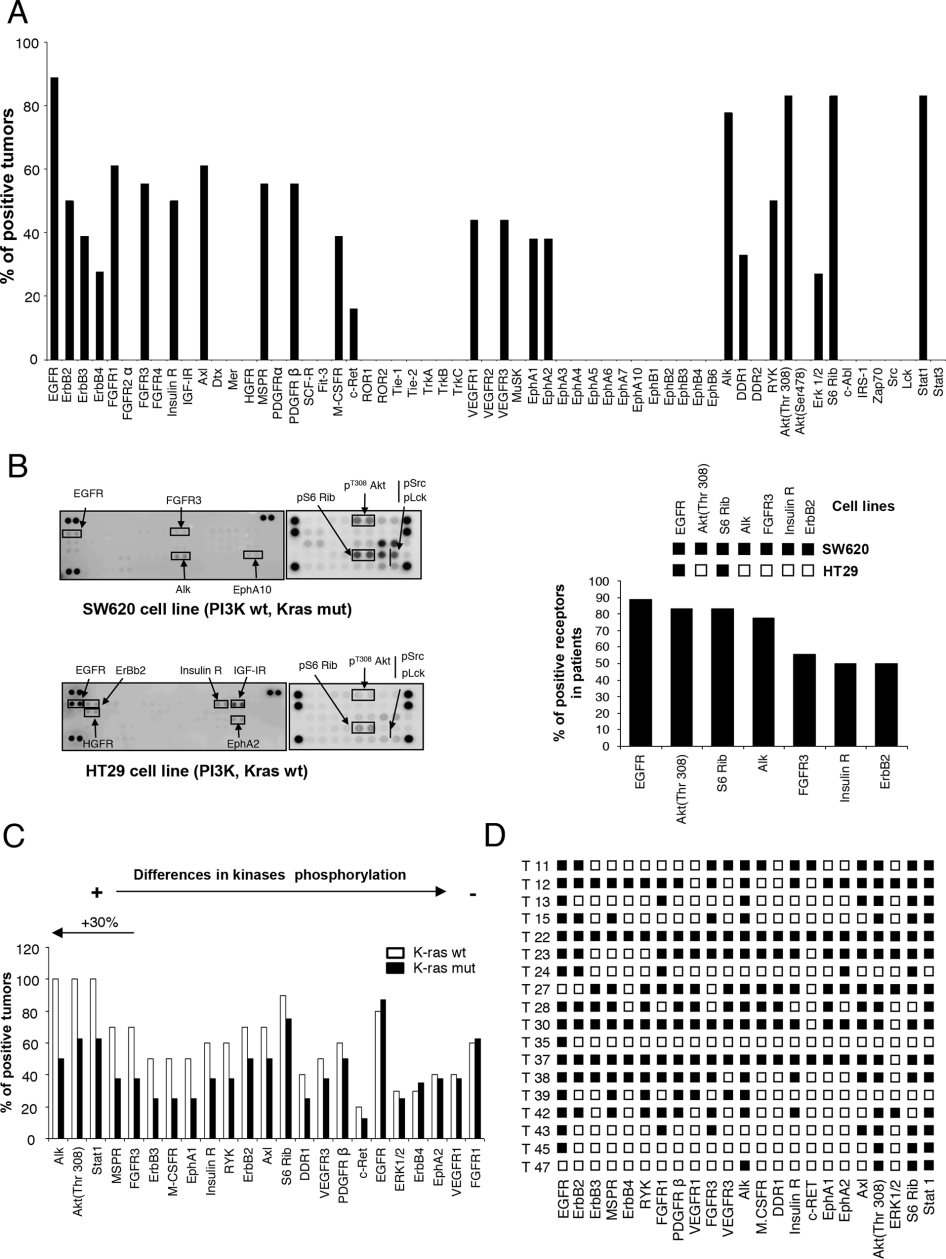
Expression of activated forms of RTKs and signaling mediators in human samples of colon cancer **A.** The histogram shows the percentage of human tumors that exhibited phosphorylated kinases. **B.** phosphorylated kinases in colon cancer cell lines, SW620 and HT29, and its comparison with phosphorylated proteins in human tumors. **C.** Relative comparison of kinases phosphorylation between tumors bearing wild-type and mutated KRAS. **D.** Expression of activated kinases in each analyzed tumor.

The analyses revealed that of the fifty-nine proteins evaluated, only twenty-three were phosphorylated (Figure 1A). The most phosphorylated RTKs involved members of the ErbB receptor family (EGFR: 88.8%, *n* = 16; ErbB2: 50%, *n* = 9; ErbB3: 38.8%, *n* = 7; ErbB4 27.7%, *n* = 5); followed by Alk (77.7%, *n* = 14), FGFR1 (61%, *n* = 11), FGFR3 (55.5%, *n* = 10), AXL (61%, *n* = 11), PDGFR beta (55.5%, *n* = 10) or IR (50%, *n* = 9), among others. We also observed activation of VEGFR1 (44%, *n* = 8) and VEGFR3 (44%, *n* = 8). Phosphorylation of downstream signalling regulators included components of the PI3K/mTOR/AKT pathway (AKT/Thr 308: 83%, *n* = 15; pS6: 83%, *n* = 15) and STAT1 (83%, *n* = 15) (Figure 1A). Next, we correlated the expression of RTKs and downstream mediators observed in human samples with those of two representative cell lines of colorectal cancer, SW620 and HT29. As can be seen in Figure 1B, some of the most activated RTKs and downstream mediators in human samples were also activated in SW620 or HT29, confirming that these cell lines can be considered as a good representative model.

Given the fact that selection of anti-EGFR therapies is based on the presence of K-RAS mutations and that tumors with constitutive activation of downstream mediators can present secondary activating loops, we interrogated if differences in the kinase profile among the two groups could be identified. Therefore, we compared the kinase profile in K-RAS mutated (*n* = 8) versus non-mutated (*n* = 10) tumors. Expression of EGFR was similar in both groups, but ALK, AKT/Thr308 and STAT1 were reduced in tumors with K-RAS mutations (Figure 1C). No differences were observed for the expression of pErk1/2. Other kinases whose phosphorylation was reduced in K-RAS mutated tumors included MSPR, FGFR3 and ErbB3 (Figure 1C).

Finally, we observed that an important number of proteins were phosphorylated within the same tumor (Figure 1D), supporting the idea that targeting of several proteins or key signalling nodes could be a rational approach.

### Pharmacologic evaluation with multi-kinase inhibitors

**Figure 2:**
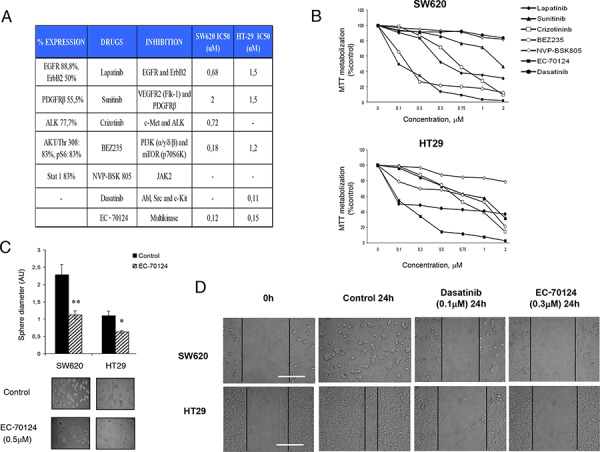
Pharmacologic screening and effect of EC-70124 on proliferation and migration **A.** List of drugs assayed in the study, including EC-70124, a novel multikinase inhibitor. Table shows the target proteins and IC50 values in SW620 and HT-29 colon cancer cell lines. **B.** Effect of the different kinase inhibitors on the MTT metabolization of SW620 and HT-29 cell lines. Cells were plated in 48-multiwell plates and treated with the indicated concentration of the drugs for 72 h. **C.** Action of EC-70124 on the morphology of SW620 and HT-29 grown in three-dimensional (3D) cultures. Cells were plated in 48 multiwell plates and grown in medium containing matrigel for 7 days in the presence of EC-70124 (500 nM). All images were taken at ×20 magnification. The quantification of sphere diameter was performed manually by tracing a straight line across the sphere diameter of untreated cells (controls) and scoring its value as arbitrary length units. Data are represented as the mean ± s.d of triplicate experiments. Student's test was used to calculate statistical significance: *P < 0.05 and **P < 0.005. **D.** Effect of EC-70124 on wound-healing process in colon cancer cells. SW620 and HT-29 were treated with EC-70124 at 300 nM and photographs were taken at 24 hours. Treatments with Dasatinib at 100 nM were used as a negative control of migration. Scale bar represents 500 μm.

Next, we decided to evaluate the effect on cell proliferation of several kinase inhibitors designed against the most frequently phosphorylated kinases observed in human samples. We evaluated six different agents, including some agents approved in cancer for other indications and a multi-kinase inhibitor currently in preclinical development. The agents included lapatinib, as an EGFR and ErbB2 inhibitor, sunitinib as a VEGFR2 and PDGFRβ inhibitor, crizotinib as a c-MET and ALK inhibitor, dasatinib as a Abl, SRC and c-Kit inhibitor, BEZ235 as a dual pan-PI3K/mTOR inhibitor, and NVP-BSK805 as a JAK/STAT inhibitor (Figure 2A). In addition, we evaluated a novel polypharmacology kinase inhibitor termed EC-70124, a hybrid indolocarbazole obtained by combinatorial biosynthesis of Rebeccamycin and Staurosporin genes [10].

The effect on cell proliferation of these compounds was evaluated in two colon cancer cell lines SW620, and HT29 using the MTT metabolization assay. By doing a dose response curve we observed different sensitivity to the drugs evaluated. The proliferation assays showed that the new multi-kinase inhibitor EC-70124 had a strong effect in the cell lines studied compared with other agents. EC-70124 reached a half-maximal inhibitory effect in the nanomolar range (below 200 nM) in the two cell lines (Figure 2A, 2B). At doses below 500 nM only BEZ235 showed a relevant effect on growth inhibition in SW620, but limited in HT29. Dasatinib showed only antiproliferative effect in HT29.

We also investigated the effect of EC-70124 in three-dimensional growth using the same cell lines. For this purpose, we grew cells in matrigel, a semisolid media where the cells grow forming spherical structures. Treatment with EC-70124 strongly decreased the diameter of these spheres (control vs treatment, mean diameter and SD = 3.62 +/− 0.11 vs 2.28 +/− 0.08 and 10,63 +/− 0.7 vs 1.1 +/− 0.1 for SW620 and HT-29, respectively) (Figure 2C).

Finally, we evaluated the effect of the drug on cell migration. EC-70124 reduced the migration of the two cell lines at 24 hours (Figure 2D).

### Effect of EC-70124 on the kinase profile of colon cancer cell lines

**Figure 3:**
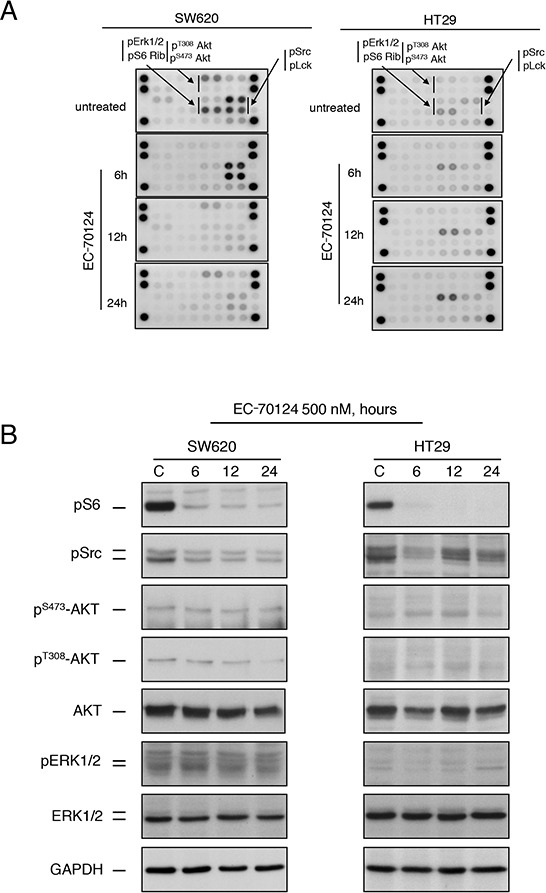
Effect of EC-70124 on RTKs and downstream signaling mediators **A.** SW620 and HT-29 cells treated with either EC-70124 (500 nM) or drug vehicle for 6 h, 12 h and 24 h were processed for protein extraction. Cell lysates containing 150 μg of total protein were analyzed for the expression of phosphorylated proteins by using an antibody array kit. **B.** Colon cancer cell lines were cultured in the presence of EC-70124 (500 nM) for the indicated times and cell lysates were analyzed by western-blot for the levels of different proteins as well as their phosphorylated forms.

To further evaluate the effect of EC-70124 on the kinase profile, we analyzed the phosphorylation status of several kinases in SW620 and HT29 before and after treatment with the drug at 6, 12 and 24 hours. A common pattern for these cell lines was the phosphorylated presence of key signalling nodes including the PI3K/mTOR pathway (AKT and S6) and SRC (Figure 3A, 3B), and as described in Figure 1B. In SW620, SRC and LCK were strongly activated in basal conditions and treatment with EC-70124 caused a strong and maintained inhibition. pS6 showed a relevant basal phosphorylation in SW620 and HT29 that was inhibited at short times. In HT29, pS6 and SRC which showed the highest phosphorylated level among all kinases, were also inhibited. In this cell line an increasing activation of Erk1/2 pathway was observed after 6 hours of treatment (Figure 3A and 3B).

### EC-70124 produces G2/M arrest and induction of apoptosis

**Figure 4:**
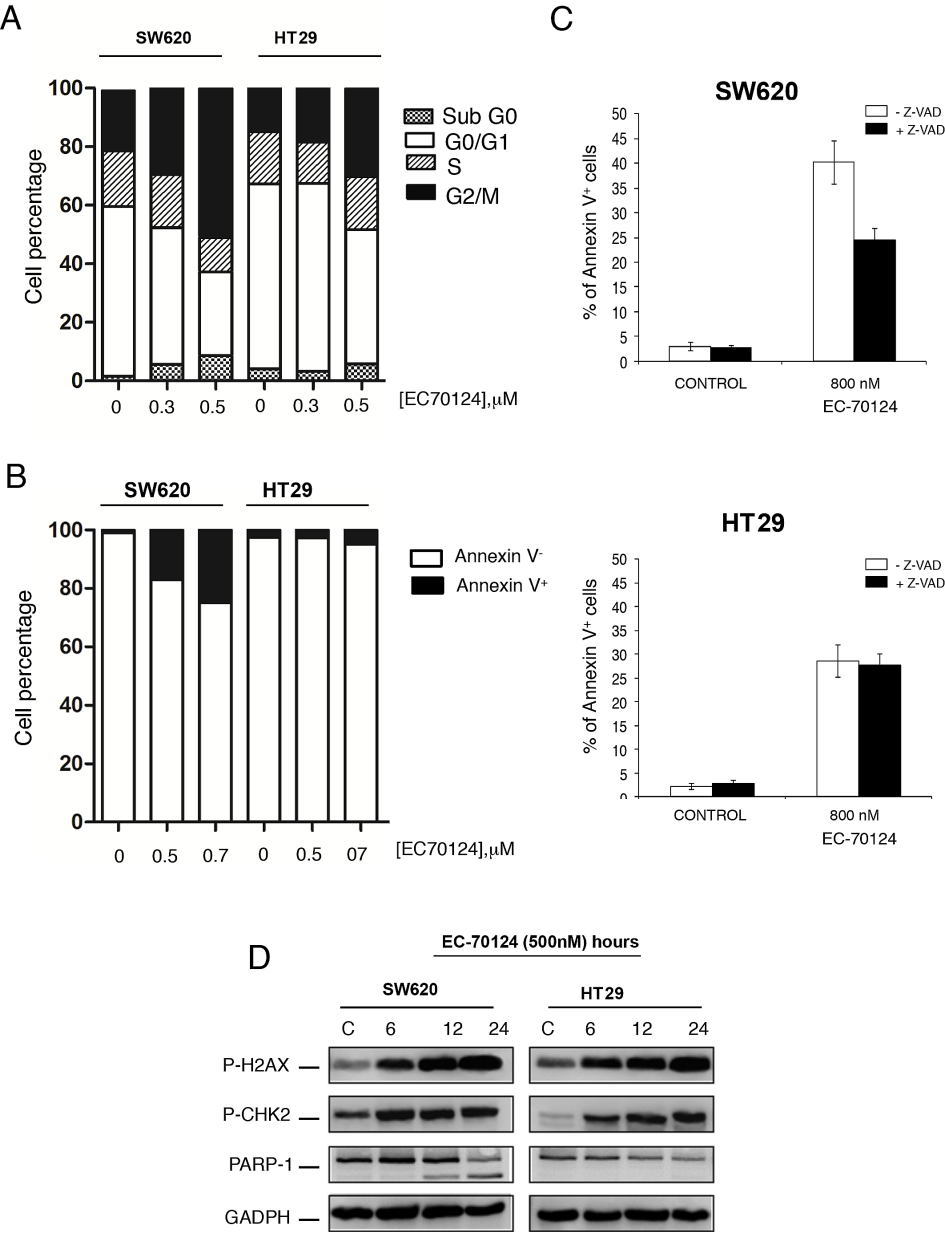
Effect of EC-70124 on cell cycle and induction of apoptosis **A.** Effect of EC-70124 on the cell cycle of SW620 and HT-29. Cells were cultured in DMEM or RPMI + 10% FBS + 5 mM glutamine and treated with 300 nM and 500 nM of EC-70124. Cell cycle progression was examined after 24 hours of treatment by flow cytometry using propidium iodide DNA staining. The histogram shows the percentage of cells in different phases of the cell cycle. **B, C.** Apoptotic effect of EC-70124. B) The histogram represents the percentage of cells positive and negative for Annexin V staining. C) Cells were treated with EC-70124 in the presence or absence of the pancaspase inhibitor Z-VAD-FMK for 48 h, and then stained with Annexin V-DY-634 and propidium iodide. Stained cells were analyzed by flow cytometry. **D.** The levels of p-H2AX, p-CHK2 and cleaved PARP of colon cell lines treated with EC-70124 at different time point were determined by Western-blot. GAPDH was used as a loading control.

Next we explored the effect of EC-70124 on cell cycle and apoptosis. Propidium iodide staining revealed that EC-70124 induced accumulation of cells in the G2/M phase (Figure 4A). Using Annexin V staining we evaluated the induction of apoptosis. As can be seen in figure 4B, treatment with EC-70124 induced apoptosis only in SW620, that was confirmed by the biochemical evaluation of PARP cleavage (Figure 4D). No such effect was observed in HT29 (Figure 4B and 4D). To explore the mechanism of cell death we treated both cells with the pancaspase inhibitor Z-VAD-FMK and evaluated Annexin V staining at 48 hours. No modifications in apoptosis was found with the presence of the pancaspase inhibitor in HT29, and a small induction in SW620 (Figure 4C). This data shows that in SW620 induction of apoptosis was both, dependent and independent of caspases.

In a previous evaluation of EC-70124 in breast cancer, we observed that EC-70124 led to DNA damage [11]. To explore if this effect was also observed in colon cancer, we assessed the accumulation of pH2AX and p-chk2 at different time points in SW620 and HT29. Treatment with EC-70124 augmented the levels of these proteins at different time points suggesting that the drug caused DNA damage in colon cancer cells (Figure 4D).

### EC-70124 synergizes with standard of care chemotherapy

**Figure 5:**
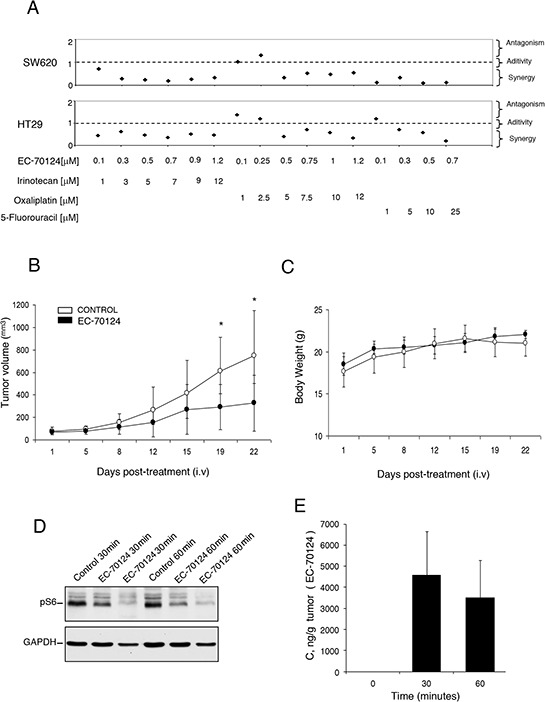
Combinational studies *in vitro* and *in vivo* antitumor action of EC-70124 **A.** SW620 and HT-29 cells were treated at the indicated concentrations of EC-70124, Irinotecan, Oxaliplatin and 5-Fluorouracil, alone and in combination, and their MTTs values were calculated. Combination indexes for the different drug combinations were obtained using CalcuSyn program and plotted. **B.** Nude mice were injected with HT-29 colon cancer cells. When tumors reached 100 mm^3^ animals were treated intravenously with either drug vehicle (control) or EC-70124 (18 mg/kg) for 3–4 days. Data represent the mean +/− s.d. *P < 0.05. **C.** Body weights curves of untreated and treated animals. **D.** Effect of EC-70124 on expression of p-S6 in mice tumors. Animals were treated for 30 and 60 minutes with EC-70124 (18 mg/kg iv) and activation of the indicated proteins were analyzed by Western-blotting. GAPDH was used as a loading control. **E.** EC-70124 accumulation in xenografted tumor at 30 min and 60 min: 4 nu/nu mice implanted with HT-29 were treated intravenously with 18 mg/Kg EC-70124 and two tumors per time point were processed for LC-MS analysis.

As success in cancer therapy is based on drug combinations, we investigated the effect of EC-70124 in association with chemotherapies used in the clinical setting for the treatment of metastatic colorectal cancer including irinotecan, 5-fluorouracil and oxaliplatin. To identify synergistic interactions we used the Chou-Talalay algorithm [12] for combination index analysis in SW620 and HT29, at different concentrations (Figure 5A). Combinations with irinotecan, oxaliplatin and 5-fluorouracil were synergistic in the two cell lines, at almost all evaluated doses (Figure 5A). Studies with clonogenic assays confirmed the increased activity of the combinations compared with each agent given alone (Supplementary Figure S2A).

Finally, we explored the biochemical mechanism of action associated with the combination of EC-70124 and chemotherapies. We observed how EC-70124 induced apoptosis when combined with chemotherapy mainly in SW620 (Supplementary Figure S2B).

### Effect of EC-70124 on tumor growth *in vivo*

Finally, to evaluate the effect of EC-70124 *in vivo*, we implanted HT29 in mice and treated animals with EC-70124 at a dose of 18 mg/kg i.v. every three days. Figure 5B shows the anti-tumor effect of EC-70124 compared to control. The mean tumor volume (mm^3^) measured every three days was significantly higher in the control versus the treated group (day 21 after treatment; control vs treated, mean volume 749.43 vs 248.44 mm^3^, SD +/− 401.4 and +/− 248.44, respectively; *p* = 0.029). No differences in body weight were observed among treated and control groups (Figure 5C). In parallel we studied the pharmacodynamic effect of EC-70124 on implanted tumors at different time points. As can be seen in Figure 5D treatment with the drug reduced pS6 at different time points (30 and 60 min), confirming the effect of this drug on this pathway. Finally, as can be seen in Figure 5E, there was a clear accumulation of the drug in the tumor at 30 min and 60 min, reaching over 3000 ng/g, well above levels with anti-proliferative activity in cell lines.

## DISCUSSION

Protein kinases are implicated in different cellular functions linked with oncogenic transformation [13]. In solid tumors several of them are deregulated and potentially druggable with kinase inhibitors [13, 14]. In the present work we aimed to explore the kinase profile of colon cancer using human samples. We observed several activated proteins including the ErbB receptor family and particularly EGFR, components of the PI3K/mTOR route including AKT and pS6, and the JAK/STAT pathway represented by STAT1, among others.

EGFR is a well known activated kinase in many epithelial tumors including colorectal cancer [15]. Indeed, strategies to inhibit this receptor with antibodies or small tyrosine kinase inhibitors have reached the clinical setting [15]. Examples are cetuximab or panitumumab approved for several indications including colorectal or head and neck tumors, or EGFR-directed small kinase inhibitors in lung cancer [3, 15, 16]. As EGFR activates downstream pathways such as the PI3K/AKT/mTOR or the MAPK route, we also explored key intermediates of these nodes. In addition, activation of the PI3K/mTOR pathway is associated with cancer and strategies aimed to neutralize their activation are in clinical development in different solid tumors [17, 18]. In our study we observed the phosphorylation of S6 and AKT/Thr308 in a high proportion of patients, showing the relevance of this route in colorectal tumors.

Ongoing studies are evaluating agents against the PI3K/mTOR route in combination with anti-EGFR antibodies in colon cancer [19]. Interestingly, phosphorylation of intermediates like AKT and S6 were less prominent in those tumors with K-RAS mutations suggesting that strategies against the PI3K/mTOR pathway should focus on K-RAS wild type tumors. Indeed, our study confirm this strategy, that has been incorporated in some clinical studies [19].

An interesting finding was the significant expression of phosphorylated ALK and STAT1 in the analyzed human samples. In colon cancer, rearrangements of ALK have been previously described in a small proportion of tumors [20] and the JAK/STAT pathway has been associated with the initiation and maintenance of cells with stem cell properties [21].

Of note, concomitant activation of several kinases were observed within the same tumor. This is a remarkable observation as suggests that drugs with activity against several relevant routes could have more antitumor activity than drugs targeting single proteins.

Next we evaluated different compounds against the most frequent phosphorylated kinases. We observed how the novel multi-target kinase inhibitor EC-70124 showed a higher antitumor activity, compared with other inhibitors. Biochemical evaluation of the mechanism of action of EC-70124 showed that this drug inhibited relevant routes including the PI3K/AKT/mTOR pathway. Furthermore, EC-70124 inhibited SRC and reduced migration of cancer cells. EC-70124 exerted its antineoplastic action through an arrest in G2/M and an induction of apoptosis, as indicated by accumulation of PARP cleavage in SW620 [22].

Additional experiments demonstrated that EC-70124 led to DNA damage. This was confirmed by phosphorylation of γH2AX and chk2. γH2AX is phosphorylated at sites of DNA damage and it is required for the recruitment of multiple DNA repair proteins [23]. Similarly, after DNA damage chk2 is phosphorylated by ATM/ATR kinases.

Most current therapeutic strategies in oncology are based on drug combinations, therefore identification of synergistic interactions between targeted agents and chemotherapies is a main goal [24]. Therefore we decided to evaluate EC-70124 in association with relevant drugs used in the clinic including oxaliplatin, 5-fluorouracil and irinotecan. EC-70124 in combination with these drugs showed a synergistic interaction in the cell lines studied. We observed how the administration of these combinations induced apoptosis particularly in SW620.

Finally, we observed that EC-70124 inhibited tumor growth in xenografted mice demonstrating its efficacy *in vivo*. Of note, a reduction of pS6 was also observed confirming the effect of the drug on the PI3K/AKT/mTOR route.

In conclusion, in this study we describe the kinase profile of human colorectal tumors, reporting some druggable kinases and identifying a novel compound against some of the activated proteins with clear antineoplastic effect. EC-70124 induces cell cycle arrest at G2/M and apoptosis. We identify combinations with chemotherapies that have a synergistic effect and confirm the activity *in vivo*. Globally, our data support the future clinical development of this drug in this clinical setting.

## MATERIALS AND METHODS

### Patient samples

Colon cancer samples were obtained from the tumor bank of the Albacete University Hospital following institutional guidelines. All patients signed the study consent form. Frozen tissues were collected from 18 randomly selected patients who had undergone surgical resection of their colorectal tumor. Before processing the samples, cancer and benign areas were separated under microscopy analysis.

### Cell lines and drug compounds

SW-620 cell line was obtained from the American Type Culture Collection (ATCC; CCL-227) (Manassas, VA) and HT-29 cell line was kindly provided by Dr. R. Sánchez-Prieto. These cells display several genetic alterations; SW-620 carries mutation in Kras gene, whereas HT-29 has a coexisting activating mutation in PIK3CA and BRAF. Cell lines were maintained in RPMI (SW620) and DMEM (HT-29) containing 10% fetal bovine serum (FBS), with 100 U/mL penicillin, 100 μg/mL streptomycin and 5 mM L-glutamine. The cell culture medium and supplements were obtained from Sigma Aldrich (St. Louis, MO). The multi-kinase inhibitor EC-70124 was prepared via a proprietary process with Entrechem S.L. (Oviedo, Spain). Chemotherapeutic agents (Irinotecan, Oxaliplatin and 5-Fluorouracil) were purchased from Selleckchem (Deltaclon, Madrid Spain).

### Preparation of tumor homogenates and phospho-kinase antibody arrays

The tumor samples of patients were minced, washed with phosphate-buffered saline buffer (PBS), and homogenized in ice-cold RIPA lysis buffer containing 10X protease and phosphate inhibitor cocktails (1.5 ml/100 mg of tumour). These homogenates were centrifuged at 10000 g for 10 min at 4°C, and the supernatants were transferred to new tubes. For preparation of EC-70124 treated and non-treated cell extracts, cell cultures were washed with cold PBS and lysed in ice-cold RIPA lysis buffer containing 1X protease and phosphate inhibitor cocktails. Then, insoluble material was removed by centrifugation. The protein concentration of tumor homogenates and cell lines extracts was determined using BCA (Bicinchoninic acid) protein assay kit (Sigma Aldrich).

The phosphorylation status of a wide range of Receptor Tyrosine Kinase (RTK) and downstream signaling nodes was evaluated in both colon cancer tissues and EC-70124-treated and non-treated cell lines. For these studies, two commercial arrays were used; the human phospho-RTK array kit (# ARY001, R&D Systems, Abingdon, UK) and the PathScan RTK Signaling Antibody Array Kit (# 7982, Cell Signaling Technology). According to the manufacturer's instructions, antibody array membranes were incubated with 1,5 mg and 150 μg of protein lysates, respectively. Quantification of the different RTKs and cell signaling intermediates in the membranes was performed using the Quantity One analysis software (Bio-Rad, CA, USA) and expressed as the pixel density (OD/mm^2^). The pixel density of the background was subtracted from the pixel density of each spot, and the average of duplicate spots was determined. Next, normalized signal intensity was calculated by dividing the mean value of pixel density in each spot by the mean value of pixel density in the positive control. Significance was determined using a cut-off point of density signal higher than 0.6.

### MTT metabolization, three-dimensional cell cultures and clonogenic assays

Cell proliferation experiments were carried out using 3-(4,5-dimethylthiazol-2-yl)-2,5-diphenyltetrazolium bromide (MTT) assays, where MTT is reduced to purple formazan by the mitochondria of living cells. Increase in cell number is detected by augmented MTT metabolization, and decrease in cell number is reflected by decrease in MTT metabolization. SW620 and HT29 cells were plated at a density of 10,000 cells per well in 48-multiwell plates and cultured overnight in RPMI or DMEM supplemented with 10% FBS and 5 mM glutamine The next day, cells were treated for three days with increasing concentrations of EC-70124 alone or in combination with various chemotherapeutics to plot the dose–response curves and for synergy studies, respectively. In parallel, cells were treated with increasing amounts of EC-70124 at three-time points (24 h, 48 h and 72 h of incubation) to determine the IC50 value. After drug administration, the medium was replaced with 400 μL of fresh medium DMEM without phenol red containing MTT (0.5 μg/μL) and incubated for 45 minutes at 37°C. The medium was then removed and 200 μL of dimethylsulfoxide (DMSO) were added to each well. The plate was agitated in the dark for 5 minutes to dissolve the MTT-formazan crystals. The absorbance of the samples was recorded at 562 nm (555–690) in a multiwell plate reader (BMG labtech). Results were plotted as the mean values of quadruplicates from a representative experiment that was repeated at least two independent times.

To determine whether EC-70124 combined to other chemotherapy drugs (Irinotecan, Oxaliplatin or 5-Fluorouracil) was synergistic, additive, or antagonist, we used the CalcuSyn v2.0 software programme (Biosoft, Ferguson, MO). This program allows the calculation of the combination index based on the algorithm of Chou and Talalay [12]. Combination index values greater than 1 indicate antagonism, less than 1 indicate synergism and values equal to 1 indicate an additive effect. Combination index values from three independent experiments were generated.

For Matrigel-embedded cell culture experiments, SW620 and HT-29 cells were grown in RPMI or DMEM supplemented with 10% FBS and 2 mM glutamine. Following passage, cells were trypsinized (0.5 g porcine trypsin and 0.2 g EDTA 4 Na, purchased from Sigma Aldrich) and resuspended in growth medium containing 2% Matrigel. Then, cells were seeded at a density of 12,500 cells/ml in a 48-multiwell plate containing an underlying approximately 1 mm thick bed of Matrigel and incubated at 37°C. Next day, cells were treated with EC-70124 and cultured for 7 days. The assay included the daily visualization of cells under a light microscope to monitor the phenotype.

For clonogenic experiments cell were seeded at a density of 500,000 cells in a 100 mm culture dishes, and treated, the next day, with EC-70124 (500 nM), Irinotecan, Oxaliplatin, 5-Fluorouracil, EC-70124 + Irinotecan, EC-70124 + Oxaliplatin and EC-70124 + 5-Fluorouracil. After 24-hours treatment, cells were trypsinized, resuspended in 5 ml of complete growth medium to perform serial dilutions 1/10 and seeded, in triplicate, in 6-multiwell plates for 10 days. Then, the medium was removed and the number of colonies were determined.

### Cell migration study

SW620 and HT-29 cells were plated at a density of 200.000 cells/60 mm dish and maintained overnight in RPMI + 10% FBS + 2 mM glutamine. Following incubation, culture medium was removed and a wound in the cell monolayers was generated by scratching with a 200-μl pipette tip. Photographs were taken of the initial wound for comparison. Then, DMEM + 10% FBS was added and cells were treated for 48 hours with either 300 nM EC-70124 or 100 nM Dasatinib, as a negative control. Cell migration was visualized at x10 magnification and photographed. Each experiment was completed in duplicate.

### Western blotting

For Western-blotting, 50 μg of total protein from cell lysates was boiled and resolved by 6%–15% sodium dodecyl sulfate polyacrylamide gel electrophoresis (SDS-PAGE), depending on the molecular weight of the proteins to be analyzed. After electrophoresis, proteins in gels were transferred to polyvinylidene difluoride membranes (Millipore Corporation). Blots were blocked in 1x Tris-buffered saline (TBS,100 mM Tris [pH 7.5], 150 mM NaCl, 0.05% Tween 20) and 1% of bovine serum albumin for 1 hour and then incubated overnight with the following primary human monoclonal/policlonal antibodies: anti-AKT, anti-p^S473^-AKT, anti-pH2AX, anti-pCHK2 (BD Biosciences), anti-ERK1/2, anti-pERK1/2, anti-PARP-1 (Santa Cruz Biotechnology), anti-pS6, anti-p^T308^-AKT (Cell Signalling Technology), anti-Src (Cell Signalling Technology). Protein-bound primary antibodies were detected using respective horseradish peroxidase-coupled secondary antibodies (anti-rabbit for polyclonal and anti-mouse for monoclonal, obtained from Santa Cruz Biotechnology) diluted 1:5,000 in 1x TBS containing 0.05% Tween and incubated for 1 hour at room temperature. Protein bands were detected using ECL Plus Western Blotting Detection System (GE Healthcare, Buckinghamshire, United Kingdom).

### Cell cycle, and apoptosis detection assays

For cell cycle analyses, SW620 and HT29 cells were plated at a density of 500,000 cells/100 mm dish and maintained overnight in DMEM + 10% FBS. Cells were then treated with 500 nM of EC-70124 for 24 hours. After drug treatment, cells were trypsinized, fixed in ice cold 70% ethanol for 30 minutes and subsequently centrifuged at 6000 rpm for 5 minutes. Cell pellets were washed in PBS + 2% BSA and treated with Propidium iodide/RNAse staining solution (Immunostep S.L., Salamanca, Spain) in the dark for 1 hour at 4°C, and analyzed then on FACSCanto II flow cytometer (BD Biosciences). The percentage of each cell cycle phase was determined by plotting DNA content against cell number using the FACS Diva software.

For apoptosis analyses, SW620 and HT29 cell monolayers were incubated in trypsin–EDTA, washed twice with cold PBS, and then resuspended in Annexin V binding buffer (Immunostep S.L., Salamanca, Spain) at a concentration of 1 × 10^6^ cells per mL. A total of 3 × 10^5^ cells were incubated for 1 hour in the dark with Annexin V (Immunostep) and PI staining solution (5 μL Annexin V-DY-634, 3 μL of PI [10 mg/ml final concentration], 400 μL binding buffer). The apoptotic cells were determined using a FACSCanto II flow cytometer (BD Biosciences). Both early apoptotic (Annexin V-positive, PI-negative) and late (Annexin V-positive and PI-positive) apoptotic cells were included in cell death determinations.

### *In vivo* studies

Animal care complied with the Principles of Laboratory Animal Care and Guide for the Care and Use of Laboratory Animals. HT-29 human colon carcinoma cells (5 × 10^6^ cells in 100 μl of DMEM with 20% Matrigel) were injected subcutaneously through a 23-gauge needle into the back of female BALB/cAnNR1-Fox1 nu/Fox 1 nu mice (5 weeks old, body weight ∼ 18 g) obtained from Janvier Labs. Mice were randomly divided into two groups: (i) drug treatment group (*n* = 5) and (ii) control group (*n* = 5). Once tumors reached a volume of 100 mm^3^, animals were inhalatorily anaesthetized and treated with either EC-70124 i.v (18 mg/kg) or vehicle i.v. (following manufactures instructions) at intervals of 3 to 4 days. Tumor diameters were recorded every 3–4 days. The results are expressed as tumor volumen, which were calculated using the following formula: V = (L × W^2^)/2, where V = volume (cubic millimeters), L = length (millimeters) and W = width (millimeters). Mice were killed by CO_2_ inhalation.

## SUPPLEMENTARY FIGURES


