# Supplementary material for: Phospho-kinase profile of colorectal tumors guides in the selection of multi-kinase inhibitors
Source: Oncotarget. 2015 Sep 22;6(31):31272–83. doi: 10.18632/oncotarget.5211 (PMC4741604; doi:10.18632/oncotarget.5211)
Supplement: Supplementary file 1 [file oncotarget-06-31272-s001.pdf]

SUPPLEMENTARY FIGURES

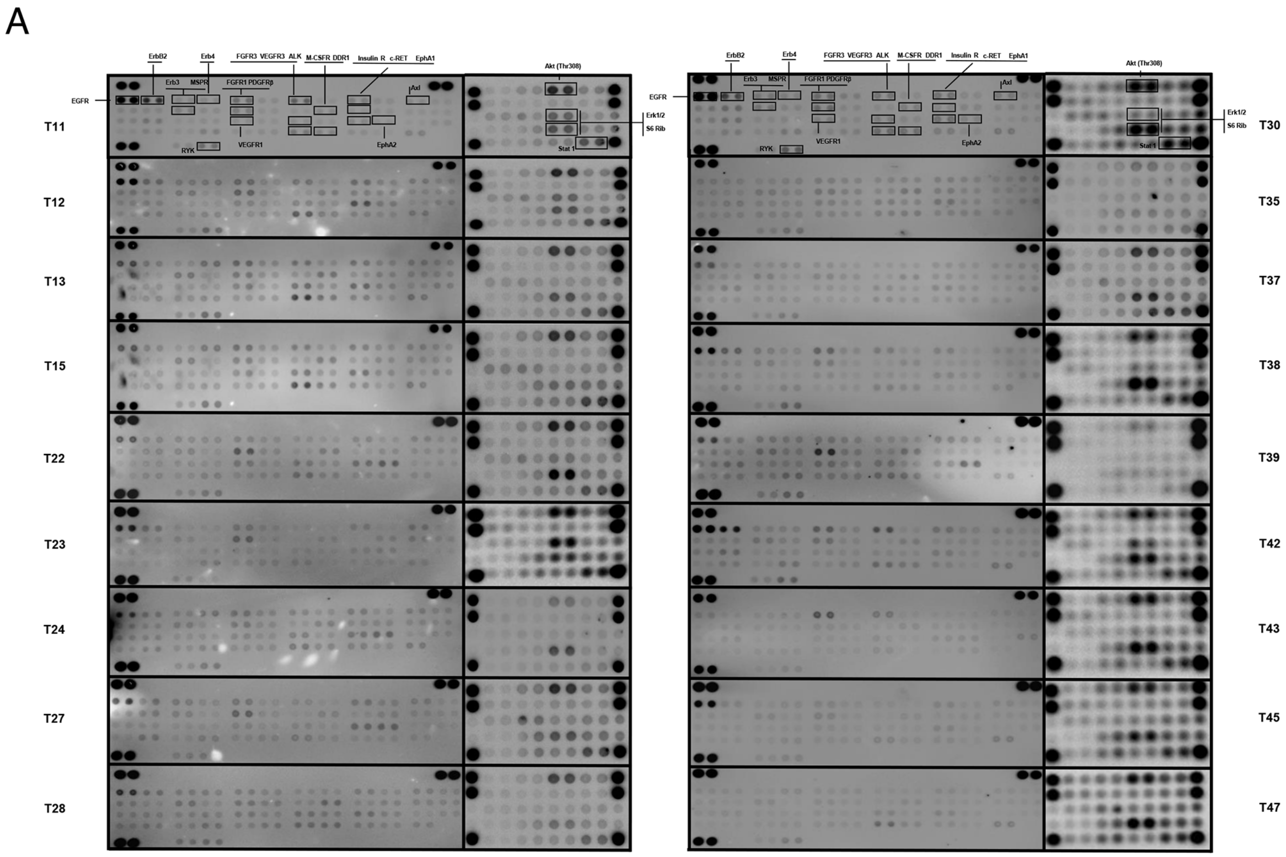

Supplementary Figure S1: Phospho-RTKs and signaling nodes array analysis. Human samples of 18 colon cancer patients were processed for protein extraction. Lysates were analyzed for the phosphorylated levels of different Receptor Tyrosine Kinases and intracellular mediators using antibody membrane kits.

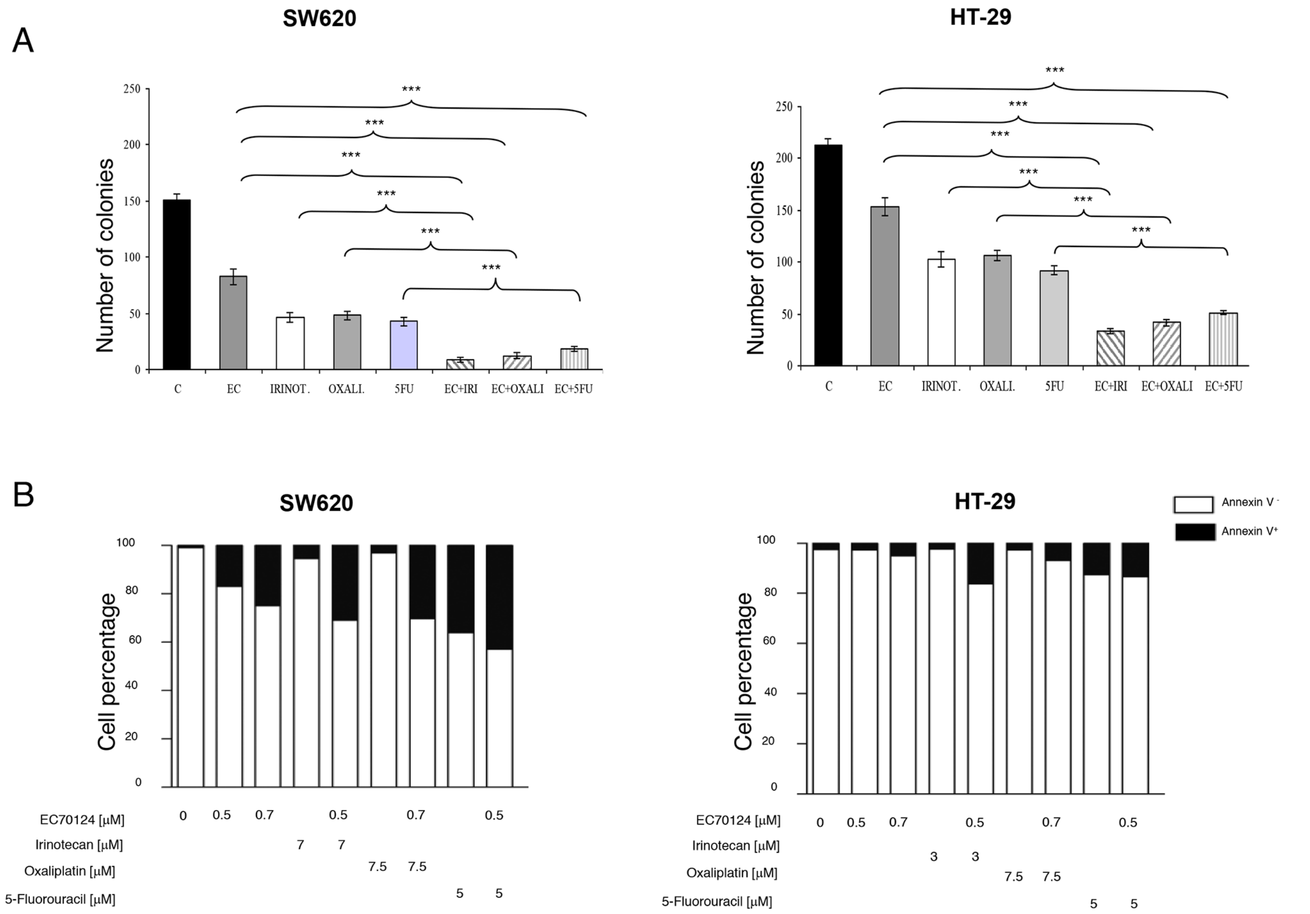

Supplementary Figure S2: Effect of the combination of EC-70124 and standard of care drugs. A. Effect on colony formation of EC-70124 alone or in combination with chemotherapies. Colon cancer cells were cultured in the presence of EC-70124 (300 nM), Irinotecan (7 µM), Oxaliplatin (5 µM) and 5-Fluorouracil (10 µM) alone and combined for 5 hours and number of colonies was determined following 10 days of treatment. The histogram represents the number of colonies formed in relation to untreated cells. Error bars indicate the mean ± s.d of quadruplicate determinations. Significant difference between treatments groups (drug alone or combined) was found (Student's test; $***P < 0.001$). **B.** Induction of apoptosis by EC-70124, Irinotecan, Oxaliplatin and 5-Fluorouracil therapies alone or in combination in SW620 and HT-29. Cells were treated with the indicated dosis of EC-70124 Irinotecan, Oxaliplatin and 5-Fluorouracil and, stained with Annexin V and propidium iodide after 48 hours of drugs incubation. The histogram represents the percentage of cells positive or negative to Annexin V staining.
